# Current Evidence on Cabazitaxel for Prostate Cancer Therapy: A Narrative Review

**DOI:** 10.1111/iju.70019

**Published:** 2025-02-25

**Authors:** Kazuhiro Suzuki, Hideyasu Matsuyama, Nobuaki Matsubara, Hirotaka Kazama, Fumiko Ueno, Hirotsugu Uemura

**Affiliations:** ^1^ Department of Urology, Graduate School of Medicine Gunma University Maebashi Gunma Japan; ^2^ JA Yamaguchi Kouseiren Nagato General Hospital Yamaguchi Japan; ^3^ Department of Medical Oncology National Cancer Center Hospital East Chiba Japan; ^4^ Specialty Care, Oncology Medical, Sanofi K.K. Tokyo Japan; ^5^ Department of Urology Kindai University Faculty of Medicine Osakasayama Japan

**Keywords:** cabazitaxel, chemotherapy, docetaxel, metastatic castration‐resistant prostate cancer, metastatic castration‐sensitive prostate cancer

## Abstract

The incidence of prostate cancer (PC) has recently increased in Japan. Androgen deprivation therapy (ADT) has been a key treatment in patients with castration‐sensitive PC (CSPC); however, resistance typically emerges through multiple mechanisms, leading to metastatic castration‐resistant PC (mCRPC). Taxane‐based therapy (i.e., docetaxel, cabazitaxel) has been standard care in patients with mCRPC. New evidence supporting the addition of androgen receptor signaling inhibitors (ARSIs, e.g., enzalutamide, abiraterone) to docetaxel and ADT for patients with metastatic CSPC (mCSPC) raises questions about the role of taxane‐based therapies and their optimal sequencing, as well as how to identify patients who may benefit from taxane‐based therapy. Here we review the evidence on taxane‐based therapy, including cabazitaxel, in the treatment of PC, with a focus on clinical and real‐world evidence from Japan. Cabazitaxel has proven effective for patients with mCRPC who have a history of ARSI and docetaxel use, and it is preferable to a second alternative ARSI, as indicated in the CARD study. The safety profile of cabazitaxel (particularly, the incidence of neutropenia) can be managed through prophylactic use of granulocyte colony‐stimulating factor, as well as a lower dosage and possibly variation of the dosage interval. However, a certain dose intensity is required because neutropenia has been identified as a potential prognostic indicator for treatment effectiveness. In the ARSI era for mCSPC, evidence on mCRPC treatment sequencing is limited. A better understanding of PC biology and the collection of real‐world data is essential for effective treatment and improved safety‐benefit outcomes.

**Abbreviations**
ADT: androgen deprivation therapy
AE: adverse event
AR: androgen receptor
ARSI: androgen receptor signaling inhibitors
CI: confidence interval
CSPC: castration‐sensitive prostate cancer
ECOG PS: Eastern Cooperative Oncology Group Performance Status
G‐CSF: granulocyte colony‐stimulating factor
HR: hazard ratio
mCRPC: metastatic castration‐resistant prostate cancer
OS: overall survival
PARP: poly[ADP‐ribose] polymerase
PC: prostate cancer
PFS: progression‐free survival
PSA: prostate‐specific antigen
TTF: time‐to‐treatment‐failure

## 1 Introduction

Prostate cancer (PC) is globally the second most common cancer among men [1]. The number of diagnoses has recently increased in Japan, and PC has become the cancer with the highest incidence, with over 98,600 cases projected in 2023 [2].

In patients with castration‐sensitive prostate cancer (CSPC), the overall survival (OS) rates for localized disease treated at an early stage are high (approximately 97%–100% 5‐year relative survival); however, prognosis tends to be poorer for metastatic disease (31.1%–22.5% 5‐year relative survival) [3, 4, 5]. The main initial treatment for metastatic PC is suppression of androgen receptor (AR) signaling through chemical or surgical castration [6]. Hence, androgen deprivation therapy (ADT) is the backbone treatment for advanced CSPC; however, resistance emerges, leading to metastatic castration‐resistant PC (mCRPC).

In addition to adenocarcinomas that progress based on AR signaling, a subset of metastatic CSPC (mCSPC) exists, which may initially respond to castration but eventually progresses to mCRPC [7]. Thus, PC is a heterogeneous disease from clinical, morphological, and molecular perspectives, often presenting with topographically and morphologically distinct tumor foci. This heterogeneous phenotype suggests that multiple mechanisms may be implicated in castration‐induced clonal selection and subsequent growth of androgen‐independent clones [8]. However, the AR is likely to continue driving the development and growth of advanced PC through mechanisms such as AR amplification and hypersensitivity, mutations of AR leading to promiscuous activation, mutations in coactivators/corepressors, AR activation independent of androgens, and intratumoral and alternative androgen production [9, 10].

Androgen receptor signaling inhibitors (ARSIs, e.g., abiraterone, enzalutamide) have become key drugs in the treatment of PC, operating by a mechanism targeted to the biological nature of the disease. However, the development of these treatments raises questions about how to predictively identify patients who are at a higher risk of developing mCRPC and may benefit from intensification of treatment, including taxane‐based chemotherapy. In this review, we summarize the current evidence supporting the use of cabazitaxel, including real‐world data in Japanese patients with mCRPC, and discuss future directions.

## 2 Treatment Strategies for Metastatic PC

**FIGURE 1:**
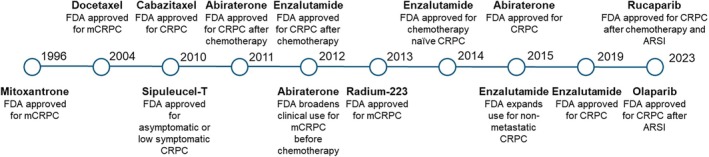
Timeline demonstrating the evolution in the treatment regimen for PC. ARSI, androgen receptor signaling inhibitors; FDA, U.S. Food and Drug Administration; mCRPC, metastatic castration‐resistant prostate cancer.

ADT has long been the cornerstone of treatment for patients with PC; however, the treatment landscape has evolved. Additional options include taxane‐based chemotherapies (docetaxel and cabazitaxel), poly[ADP‐ribose] polymerase (PARP) inhibitors (rucaparib, olaparib), immunotherapies (sipuleucel‐T), ARSIs, and radiotherapies (radium‐223) (Figure 1) [11, 12]. Appropriate selection of treatment options for metastatic PC depends on multiple factors, such as the treatment goals (curative/prolongation of OS or management of symptoms), the presence of comorbidities, and the stage and risk profile of the disease [13].

### 2.1 Risk Stratification

The National Comprehensive Cancer Network (NCCN) Clinical Practice Guidelines in Oncology currently recommend stratification of clinically localized disease into very low, low, intermediate, high, and very high on the basis of clinical features, including prostate‐specific antigen (PSA) levels, Gleason pattern, and imaging evaluations [14].

While this risk stratification is crucial for managing localized disease, it is important to note that the landscape changes significantly in the context of mCSPC. The distinction between synchronous (de novo) and metachronous (relapsed) mCSPC has been widely recognized, along with the importance of disease volume in this setting, as defined by the CHAARTED criteria (the presence of visceral metastasis or ≥ 4 bone lesions with ≥ 1 beyond the vertebral bodies and pelvis) [15]. The recurrence of dormant PC stems from its heterogeneity, driven by disseminated cancer cells that migrate early and remain dormant before activating to form metastases. Targeting these cells in earlier disease states represents a crucial window of opportunity to improve patient outcomes [16].

### 2.2 Current Guidelines and Consensus for Metastatic PC

A consensus, including physicians from across the US, Europe, and Japan, recommends adding ARSIs to chemotherapy with ongoing ADT for patients with high‐volume mCSPC who can tolerate the combination therapy [15]. The latest NCCN Guidelines for PC also now recommend this combination first‐line treatment as an option for high‐volume de novo disease with triplet therapy, that is, ADT, docetaxel as chemotherapy, and either abiraterone or darolutamide as ARSIs [17]. Subsequent therapies for patients who progress to mCRPC may include chemotherapies (i.e., cabazitaxel), immunotherapies, and radiopharmaceuticals, depending on factors such as prior treatment exposures, patient condition, and biomarkers; however, a second alternative ARSI is not recommended.

According to the NCCN Guidelines [14], the preferred systemic therapies for mCRPC depend on subtypes of PC and prior treatments. Options for adenocarcinoma include continuation of ADT alongside docetaxel and/or ARSIs (enzalutamide/abiraterone) if the patient has not received those as prior therapies. Cabazitaxel may also be considered in cases of prior docetaxel exposure. The American Urological Association Guidelines also recommend the addition of an ARSI or docetaxel to continuous ADT in patients with mCSPC and patients with mCRPC who have not previously received ASRIs [18]. Patients with mCRPC who have previously received docetaxel may be offered cabazitaxel. The radiopharmaceutical ^177^Lu‐PSMA‐617 may also be offered for mCRPC patients who have previously received both docetaxel and an ARSI.

Current Japanese guidelines acknowledge the benefits of combining ADT with an ARSI or chemotherapy as an initial treatment for patients in the mCSPC setting [19]. However, owing to limited evidence supporting any specific optimal first‐line treatment in patients with mCRPC who have already received ARSIs and/or chemotherapy for mCSPC, the guidelines suggest that decisions be made in line with the patient's condition and preferences [19].

## 3 Taxane‐Based Chemotherapy in Metastatic PC

Taxane‐based chemotherapy is currently a key component of therapy for advanced PC since the early 2000s [20]. In the pivotal TAX‐327 phase III trial, patients treated with docetaxel experienced a significant improvement in OS compared with those treated with mitoxantrone (hazard ratio [HR] 0.83, 95% confidence interval [CI] 0.70–0.99, *p* = 0.04) and therefore, docetaxel has become the preferred first‐line therapy for mCRPC [21]. Docetaxel induces microtubule stabilization, mitotic arrest, and apoptotic cell death and may also inhibit transcriptional activation of AR target genes such as PSA [22, 23]. However, patients receiving docetaxel may develop resistance associated with signal transduction pathways, such as inactivation of persistently phosphorylated AKT [24].

Cabazitaxel is a second‐generation taxane and a semisynthetic, structurally related analogue of docetaxel, developed based on its effectiveness in docetaxel‐sensitive and docetaxel‐resistant cell lines [25, 26, 27]. Cabazitaxel was developed to address a key mechanism of resistance to docetaxel, namely, the molecule's high affinity for drug‐resistance proteins, including the ATP‐dependent drug efflux pump P‐glycoprotein [27]. Cabazitaxel, in combination with prednisone, is approved for the treatment of mCRPC in patients previously exposed to docetaxel.

After the establishment of the efficacy and safety of taxane‐based therapy for mCRPC, the upfront use of docetaxel in mCSPC was evaluated in the GETUG‐AFU15 trial. This trial evaluated the addition of docetaxel to ADT in patients with mCSPC and showed no difference in OS between groups who received ADT and ADT with docetaxel [28]. However, the later STAMPEDE and CHAARTED trials have both reported benefits to OS in patients with mCSPC who received docetaxel with ADT compared with those who received ADT alone [29]. The addition of docetaxel to ADT is considered to be better suited to patients with mCSPC who have a poorer prognosis, including a high volume of disease and a larger primary tumor [30]. Thus, to date, the standard of care for patients with mCSPC, particularly among those with a high disease burden, has been upfront docetaxel combined with ADT.

In Japan, the combined androgen blockade approach, involving intensification of ADT with the addition of a nonsteroidal antiandrogen drugs, such as bicalutamide, has also shown additional benefits in terms of progression‐free survival (PFS) and OS in such patients, compared with ADT alone [31]. However, upfront docetaxel combined with ADT has been reported to confer complete PSA response in 43.5% (20/46) of Japanese patients with mCSPC [32]. In the ENZAMET trial, which assessed enzalutamide versus standard nonsteroidal antiandrogen drugs in combination with ADT with and without docetaxel, patients with mCSPC who received the enzalutamide combination experienced a notable improvement in OS compared with those who received nonsteroidal antiandrogen drugs (HR 0.67, 95% CI 0.52–0.86) with comparable incidence of adverse events (AEs) (any AE: 563 and 558, respectively) [33]. The PEACE‐1 trial found that treatment with ADT, docetaxel, and abiraterone in patients with de novo mCSPC improved OS and radiographic PFS with a modest increase in toxicity [34]. The ARASENS trial showed clear benefits in OS and key secondary endpoints, including time to CRPC, for triplet systemic therapy (addition of darolutamide to docetaxel and ADT) [35].

## 4 Cabazitaxel in mCRPC

The phase III TROPIC trial of cabazitaxel for the treatment of mCRPC confirmed the efficacy of cabazitaxel at 25 mg/m^2^ in patients progressing after docetaxel (Table 1) [36, 37]. Cabazitaxel at 25 mg/m^2^ was approved in Japan following pharmacokinetic and clinical studies in Japanese patients conducted after the TROPIC trial [38, 39]. The phase III PROSELICA trial assessed the noninferiority of cabazitaxel 20 versus 25 mg/m^2^ in post‐docetaxel patients with mCRPC and showed noninferiority in OS (13.4 vs. 14.5 months, HR 1.02) with a favorable safety profile [40]. The FIRSTANA trial established that cabazitaxel (20 and 25 mg/m^2^) was nonsuperior to docetaxel as a first‐line treatment for chemotherapy‐naïve patients with mCRPC; however, the lower dose of cabazitaxel was noted to offer a more favorable safety profile than the higher dose [41]. The CARD trial pointed to survival benefits from the use of cabazitaxel in patients who progressed on first ARSI within 1 year in the mCRPC setting compared with the use of a second alternative ARSI therapy, which may be less effective owing to cross‐resistance between abiraterone and enzalutamide [42, 43]. Furthermore, the TAXYNERGY trial suggested that early administration of cabazitaxel in patients with a poor initial response to docetaxel may be associated with an overall improved PSA response compared with patients remaining on docetaxel [44]. Thus, because cabazitaxel retains its activity after failure of prior docetaxel and ARSIs, it has become a viable treatment option in the current treatment landscape where docetaxel and ARSIs are shifting to earlier stages, except in cases where chemotherapy is contraindicated.

**TABLE 1:** Summary of key studies of cabazitaxel.

Study	Design	Patients	PFS	OS	Safety
Clinical trials					
TROPIC phase III [37]	Cabazitaxel (vs. mitoxantrone) in patients with mCRPC previously treated with docetaxel	Mitoxantrone, *n* = 377; cabazitaxel *n* = 378	Cabazitaxel 2.8 months (95% CI 2.4–3.0) vs. mitoxantrone 1.4 months (95% CI 1.4–1.7) (HR 0.74, 0.64–0.86, *p* < 0.0001)	Cabazitaxel 15.1 months (95% CI 14.1–16.3) vs. mitoxantrone 12.7 months (11.6–13.7) (HR 0.70, 95% CI 0.59–0.83, *p* < 0.0001)	Grade 3 neutropenia: mitoxantrone, 215 [58%]; cabazitaxel, 303 [82%]
FIRSTANA phase III [41]	Cabazitaxel (20 and 25 mg/m^2^) vs. docetaxel in patients with mCRPC at the first line	20 mg/m^2^, *n* = 389; 25 mg/m^2^, *n* = 388; docetaxel, *n* = 391	N/A	Cabazitaxel 20 and 25 mg/m^2^, 24.5 and 25.2 months, respectively, vs. docetaxel 24.3 months (HR 1.01, 95% CI 0.85–1.20, *p* = 0.997 and HR 0.97, 95% CI 0.82–1.16, *p* = 0.757, respectively)	≥ Grade 3 TEAEs: 20 mg/m^2^, 41.2%; 25 mg/m^2^, 60.1%; docetaxel, 46.0%
PROSELICA phase III [40]	Cabazitaxel 20 vs. 25 mg/m^2^ in patients with mCRPC previously treated with docetaxel	20 mg/m^2^, *n* = 598; 25 mg/m^2^, *n* = 602	20 mg/m^2^ 2.9 months; 25 mg/m^2^ 3.5 months (HR 1.099, 95% CI 0.974–1.240)	Cabazitaxel 20 mg/m^2^ 13.4 months vs. cabazitaxel 25 mg/m^2^ 14.5 months (HR 1.024)	≥ Grade 3 TEAEs: 20 mg/m^2^, 39.7%; 25 mg/m^2^, 54.5%
AFFINITY phase III [45]	Cabazitaxel vs. cabazitaxel with custirsen in patients with mCRPC	Cabazitaxel, *n* = 318; cabazitaxel with custirsen, *n* = 317	N/A	Cabazitaxel 13.4 months, 95% CI 12.1–14.9; cabazitaxel with custirsen 14.1 month, 95% CI 12.7–15.9 (HR 0.95, 95% CI 0.80–1.12, log‐rank *p* = 0.53)	≥ Grade 3 neutropenia: cabazitaxel, 22%; cabazitaxel with custirsen, 20%
CARD phase IV [42]	Cabazitaxel vs. ARSI in patients with mCRPC previously treated with docetaxel and had progression within 12 months while receiving the alternative ARSI	Cabazitaxel, *n* = 129; ARSI *n* = 126	Cabazitaxel 4.4 months; ARSI 2.7 months (HR 0.52, 95% CI 0.40–0.68, *p* < 0.001)	Cabazitaxel 13.6 months vs. ARSI 11.0 months (HR 0.64, 95% CI 0.46–0.89, *p* = 0.008)	≥ Grade 3 AEs: cabazitaxel, 56.3%; ARSI, 52.4%
CABASTY phase III [46]	Cabazitaxel 16 mg/m^2^ Q2W vs. 25 mg/m^2^ Q3W in patients ≥ 65 years with mCRPC	16 mg/m^2^ Q2W, *n* = 99; 25 mg/m^2^ Q3W, *n* = 97	N/A	N/A	≥ Grade 3 neutropenia: 16 mg/m^2^ Q2W, 62.5%; 25 mg/m^2^ Q3W, 62.5%
Phase II [47]	Cabazitaxel 25 mg/m^2^ Q3W vs. experimental drug monitoring in patients with mCRPC	25 mg/m^2^ Q3W, *n* = 40; drug monitoring, *n* = 33	25 mg/m^2^ Q3W 4.4 months vs. drug monitoring 9.5 months (HR 0.46, *p* = 0.005)	OS: Cabazitaxel 25 mg/m^2^ Q3W 7.3 months vs. drug monitoring 16.2 months (HR 0.33, *p* < 0.0001)	CFR: 25 mg/m^2^ Q3W, 69.4%; drug monitoring, 64.3% (*p* = 0.79)
Phase II [48]	[177Lu]Lu‐PSMA‐617 vs. cabazitaxel in patients with mCRPC	[^177^Lu]Lu‐PSMA‐617, *n* = 98; cabazitaxel, *n* = 85	N/A	N/A	≥ Grade 3 AEs: [^177^Lu]Lu‐PSMA‐617, 33%; cabazitaxel, 53%
Phase II [49]	Cabazitaxel 25 mg/m^2^ Q3W vs. 10 mg/m^2^ Q1W in patients with mCRPC previously treated with docetaxel	N/A	N/A	Cabazitaxel 25 mg/m^2^ Q3W median 6.0 vs. 10 mg/m^2^ Q1W median 6.4 (HR 0.73, 95% CI 0.47–1.13, *p* = 0.156)	N/A
TAXYNERGY, phase II [44]	Early taxane switch (docetaxel to cabazitaxel) in patients with mCRPC	Docetaxel, *n* = 41; cabazitaxel, *n* = 22	Composite: 9.1 months (95% CI 4.9–11.7)	N/A	N/A
Japanese studies					
Phase I [39]	Pharmacokinetic study of cabazitaxel 20 vs. 25 mg/m^2^ patients in Japanese patients with mCRPC	20 mg/m^2^, *n* = 4; 25 mg/m^2^, *n* = 13	N/A	N/A	≥ Grade 3 neutropenia: 20 mg/m^2^, 75%; 25 mg/m^2^, 100%
Phase I [38]	Cabazitaxel in Japanese patients with mCRPC previously treated with docetaxel	*N* = 44	N/A	N/A	Frequent AEs: febrile neutropenia, 54.5%; fatigue, 54.5%; nausea, 52.3%; diarrhea, 50.0%
Retrospective study [50]	Cabazitaxel in Japanese patients with mCRPC previously treated with docetaxel and 1 or 2 ARSIs	1 ARSI, *n* = 34; 2 ARSIs, *n* = 32	1 ARSI, 11.7 (range 7.8–14.3) months; 2 ARSIs, 7.9 (range 2.2–11.2) months (*p* = 0.03)	N/A	Any grade neutropenia: 1 ARSI, 64.7%; 2 ARSIs, 71.9%
Retrospective study [51]	Cabazitaxel in Japanese patients with mCRPC previously treated with docetaxel	*N* = 63	4.1 months	15.2 months	N/A
Retrospective study [52]	Cabazitaxel in Japanese patients with mCRPC	*N* = 66	N/A	9 months	N/A
Retrospective study [51]	Cabazitaxel in Japanese patients with mCRPC treated up to third line	Third‐line cabazitaxel, *n* = 81; three other treatments, *n* = 85	Third‐line cabazitaxel 4.2 months; three other treatments 2.8 months	Third‐line cabazitaxel 14.9 months; three other treatments 7.1 months	N/A
Retrospective study [53]	Cabazitaxel vs. ARSI in patients with mCRPC previously treated with docetaxel and had progression within 12 months while receiving the alternative ARSI	Third‐line cabazitaxel, *n* = 247; second third‐line ARSI, *n* = 288	N/A	N/A	TTF Third‐line cabazitaxel, 109 (94–128) days; second third‐line ARSI, 58 (57–66) days (HR 0.339, 95% CI 0.279–0.413)
Retrospective study [54]	Cabazitaxel in Japanese patients with mCRPC, including patients ≥ 75 years	*N* = 47		16.1 months	No correlation of patient age and OS (*p* = 0.537)
Questionnaire [55]	Cabazitaxel in Japanese patients with mCRPC	*N* = 55	5.0 months	13.0 months	No correlation of patient age and OS (*p* = 0.5435)
Retrospective study [56]	Cabazitaxel in Japanese patients with mCRPC, including patients ≥ 75 years	< 75 years, *n* = 50; ≥ 75 years, *n* = 50	< 75 years, 0.23 years; ≥ 75 years, 0.43 years (*p* = 0.32)	< 75 years, 0.69 years; ≥ 75 years, 1.17 years (*p* = 0.082)	≥ Grade 3 neutropenia: < 75 years, 72.0%; ≥ 75 years 75.0% (*p* = 1.00)
Retrospective study [57]	Cabazitaxel in Japanese patients with mCRPC, including patients ≥ 80 years	< 80 years, *n* = 610; ≥ 80 years, *n* = 49	N/A	< 80 years, 319 days (95% CI 296.0–NE); ≥ 80 years, 292 days (95% CI 199.0–NE)	< 80 years, 48.4%; ≥ 80 years, 42.0%
Retrospective study [58]	Cabazitaxel in Japanese patients with mCRPC after docetaxel	*N* = 146	PSA ≤ 20 ng/mL (median 12 months, 95% CI 11–16); PSA > 20 ng/mL (median 10 months, 95% CI 8–11)	PSA ≤ 20 ng/mL (median 31 months, 95% CI 22–44); > 20 ng/mL (median 18 months, 95% CI 15–25)	N/A

Abbreviations: AE, adverse event; ARSI, androgen receptor signaling inhibitor; CI, confidence interval; HR, hazard ratio; mCRPC, metastatic castration‐resistant prostate cancer; N/A, not available; NE, not evaluable; OS, overall survival; PFS, progression‐free survival; PSA, prostate‐specific antigen; Q1W, every week; Q2W, every 2 weeks; Q3W, every 3 weeks; TEAE, treatment‐emergent adverse event; TTF, time‐to‐treatment‐failure.

### 4.1 Results of Clinical Trials Versus Real‐World Evidence of Cabazitaxel

Studies of cabazitaxel in real‐world settings have typically reported tolerability profiles similar to those of clinical trials (Table 1) [59, 60, 61]. In Japan, real‐world studies and postmarketing surveillance of cabazitaxel have found the safety profile to be generally consistent with that reported in local and global clinical trials [62]. A retrospective analysis suggested that cabazitaxel treatment resulted in more favorable OS outcomes in patients when the treatment was introduced before the patients' PSA values increased substantially above their baseline at diagnosis (i.e., to approximately 150 ng/mL) [52].

## 5 Dosage Intensity, Efficacy, and Safety Management

The use of granulocyte colony‐stimulating factor (G‐CSF) is critical for safety management of cabazitaxel because of neutropenia [46, 63]. The recommended dose of cabazitaxel in patients with mCRPC is 25 mg/m^2^ but is commonly modified (typically to 20 mg/m^2^) in clinical practice [64]. The results of a postmarketing surveillance in the Japanese population indicated that time to treatment failure was slightly prolonged in the group receiving an initial dose of 25 mg/m^2^ cabazitaxel (median 113 days, 95% CI 94–137 vs. 120 days, 95% CI 104–157; odds ratio 0.78, 95% CI 0.62–0.97, *p* < 0.026); OS was better as well (median 287 days, 95% CI 240–326 vs. NR) [65].

In terms of safety, the PROSELICA [40] and FIRSTANA [41] trials, which did not allow prophylactic G‐CSF from cycle 1, reported that patients receiving cabazitaxel at a lower dose (20 vs. 25 mg/m^2^) had fewer cases of grade ≥ 3 neutropenia (PROSELICA: 41.8% vs. 73.3%; FIRSTANA: 37.8% vs. 70.6%) and febrile neutropenia (PROSELICA: 2.1% vs. 9.1%; FIRSTANA: 2.4% vs. 12.0%).

Some real‐world studies have suggested that cabazitaxel 20 mg/m^2^ may be appropriate for older patients and heavily pretreated patients with mCRPC [66]. A recent phase II trial demonstrated the potential of pharmacokinetic‐guided dosing adjustment, increasing dosage in patients as necessary to improve clinical outcomes [47]. However, post hoc analysis of the TROPIC phase III trial has suggested that the absence of neutropenia while receiving cabazitaxel is associated with poor outcomes [67, 68]. This proposal is supported by results from real‐world studies that have pointed to the incidence of neutropenia being a prognostic indicator for treatment effectiveness [69, 70].

Nevertheless, a European phase II trial has confirmed that weekly treatment of cabazitaxel at a lower dose may have a reduced incidence of febrile neutropenia compared with the standard dose and 3‐week schedule [49]. The CABASTY phase III trial of biweekly and triweekly cabazitaxel 16 mg/m^2^ in patients ≥ 65 years noted a reduced incidence of neutropenia and concluded that a biweekly regimen at the reduced dose may be more appropriate for patients considered too old for standard treatment timelines [46].

Notably, the TAX327 and VENICE trials for docetaxel, as well as the TROPIC [36, 59] trial for cabazitaxel, suggested that although neutropenia occurs more frequently with cabazitaxel than with docetaxel, the incidence of patient‐reported AEs, such as alopecia, peripheral neuropathy, nail disorders, and taste disturbances, is lower with cabazitaxel [47]. These findings are further supported by the results of the head‐to‐head FIRSTANA trial [41], which unambiguously showed that cabazitaxel has much lower rates of peripheral neuropathy (12% vs. 25%), alopecia (9%–13% vs. 39%), and nail disorders (< 1% vs. 9%) compared with docetaxel. The patient preference CABADOC trial also found that a greater number of patients preferred cabazitaxel, reporting lower fatigue and better quality of life [71].

## 6 Real‐World Evidence of Cabazitaxel in Older Patients

PC is primarily a disease affecting older men, with the median age of patients at death being 80 years [72]. The use of cabazitaxel in heavily pretreated older patients already at risk of AEs warrants special care from physicians and has particularly motivated studies of alternative dosages and intervals between cabazitaxel cycles in these patients.

The FUJI cohort of cabazitaxel in clinical practice for mCRPC in France recruited 401 patients having a median age of 70 years (interquartile range 65–77) and found that approximately half of the patients started at a lower dose (20 rather than 25 mg/m^2^) [60]. Although the OS among patients in this study was shorter than reported in prior clinical trials, the study included patients of poorer health, who would not have satisfied the enrollment criteria of the TROPIC and PROSELICA trials.

The CAPRISTANA study with real‐world patients having a median age of 69 years (range 47–87) reported survival and AE outcomes similar to those reported in clinical trials, though more patients aged ≥ 75 years were included in CAPRISTANA [59] (23.8%) than in TROPIC (19.0%–22.3%) [36, 59]. Notably, dose reductions were required in only 14.3% of patients. According to post hoc analysis of pooled registry data in Europe, including CAPRISTANA and early access programs, cabazitaxel is generally well tolerated; however, some baseline characteristics (Eastern Cooperative Oncology Group Performance Status [ECOG PS], frailty, metastatic sites, prior docetaxel treatment duration) may impact cabazitaxel treatment duration, with the data suggesting that patients with a poorer prognosis, more aggressive disease, or poor ECOG PS tended to receive fewer cabazitaxel cycles [61].

Data from Europe has suggested that patients ≥ 80 years old can still benefit from cabazitaxel with adjustment of dosage or treatment schedule to better manage AEs [73]. Another recent trial has noted that the neutrophil‐to‐lymphocyte ratio may be a predictive factor for a biweekly treatment schedule or reduced dosage to proactively manage neutropenia‐related AEs in patients aged ≥ 65 years [46].

In line with these international studies, the benefits of cabazitaxel in older patients progressing after docetaxel have also been confirmed in Japan [54, 55, 56]. A postmarketing surveillance study found that patients ≥ 80 years with mCRPC who received cabazitaxel tended to have similar treatment histories compared with their younger counterparts [57]. In the real world, fewer older Japanese patients receive the full initial 25 mg/m^2^ dose of cabazitaxel; however, the average dose per cycle was numerically similar between the age groups. The incidence of adverse drug reactions was comparable between age groups (< 80 years, 77.2%; ≥ 80 years, 79.6%), and febrile neutropenia was not notably higher among older patients (< 80 years, 48.4%; ≥ 80 years, 57.1%). These findings may be attributed to physicians' selection of the cabazitaxel dose based on their patients' status. Postmarketing surveillance of Japanese patients with mCRPC has also found baseline factors associated with treatment discontinuation, including the presence of liver lesions, poor ECOG PS, and higher PSA levels [74]. The presence of low PSA is also likely to indicate an optimal timing for the initiation of cabazitaxel [52]. Hence, these factors may be considered by physicians when deciding on an appropriate starting dosage and schedule for patients.

## 7 CARD‐Like Real‐World Evidence

The CARD trial explored the sequencing of cabazitaxel treatment in CRPC after docetaxel and ≤ 1‐year treatment with an ARSI and found that patients who received cabazitaxel at the third line rather than an alternative ARSI experienced benefits in terms of PFS [42]. Retrospective studies in Japan have also pointed to benefits for patients receiving cabazitaxel at the third line [75]. These findings are in line with a CARD‐like analysis of patient data from a postmarketing surveillance in Japan focused on time‐to‐treatment failure rather than imaging‐based PFS [53]. In this real‐world study, lowering the dosage of cabazitaxel from 25 mg/m^2^ was more common than in the CARD trial. However, the key findings of the postmarketing surveillance were similar to those of the CARD trial in that the median time‐to‐treatment failure of cabazitaxel at the third line was approximately twice that of a second ARSI (109 vs. 58 days, respectively). Thus, the effectiveness of a second ARSI at the third line is likely to be limited by cross‐resistance between ARSIs [43].

## 8 Discussion

New targeted therapies, such as PARP inhibitors, and emerging options like AKT inhibitors, together with intensification strategies, are significantly advancing the treatment landscape for mPC, and the increasing use of ARSI and/or docetaxel in addition to ADT as the treatment of choice for mCSPC has further complicated treatment optimization, raising new challenges in finding the optimal sequence of therapies [76]. The PEACE‐1 and ARASENS trials established triplet therapy as a standard‐of‐care treatment option for patients with mCSPC [34, 35]. The results of these trials suggest that chemo‐fit patients with high‐volume disease benefit from intensive triplet therapies, although there is a need for further verification of safety and effectiveness among Japanese patients.

The key lies in understanding the clinical and genetic heterogeneity of PC and leveraging risk stratification to select the most appropriate combination of available therapies [77]. Risk stratification models other than disease volume have been proposed based on combinations of various parameters. Molecular and genetic markers predict prognosis and response to specific treatments, enabling patient stratification and personalized therapy for early and advanced PC [48, 78, 79, 80, 81]. A secondary analysis of the CHAARTED trial showed that different molecular subtypes of mCSPC have varying prognoses and respond differently to chemo‐hormonal therapy [82]. ADT therapy alone in patients with the luminal B subtype has been associated with poor prognosis, which significantly improves with the addition of docetaxel to ADT (OS: HR 0.45, *p* = 0.007). In contrast, the basal subtype showed no OS benefit (HR 0.85, *p* = 0.58) even among those with high‐volume disease [82]. Other retrospective biomarker studies reported that lower expression of tumor suppressor gene (*PTEN*, *TP53*, and *RB1*) signatures and high *ARV7* and low *RB1* gene expression were associated with rapid development of resistance in receiving docetaxel and ADT in mCSPC [83]. Conversely, AR and estrogen receptor signatures and *ESR2* gene expression were associated with good prognoses in patients who receive ADT and docetaxel [83].

**FIGURE 2:**
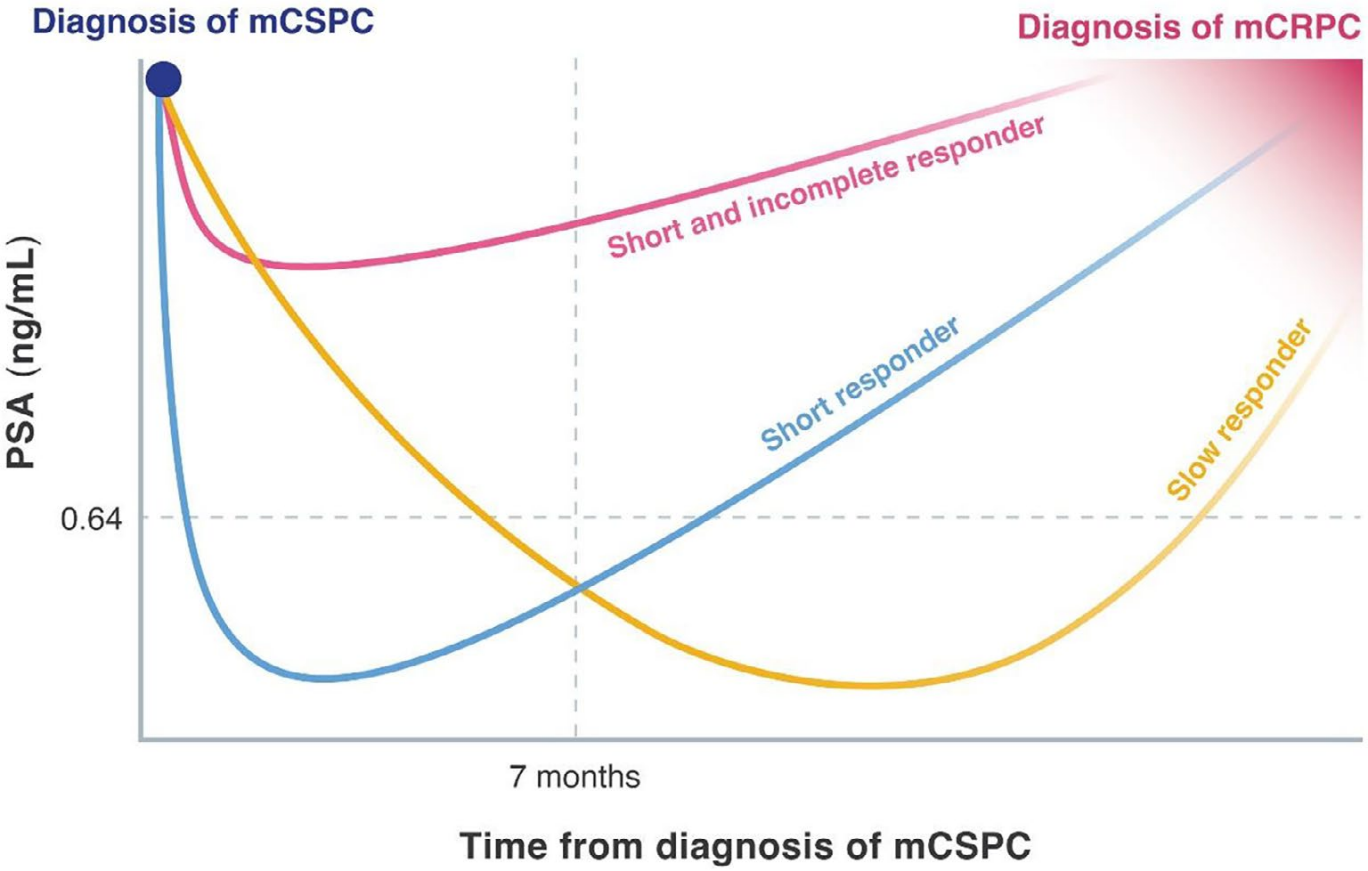
Model for heterogeneity of PSA kinetics in patients with mCSPC treated with ADT who progress to mCRPC. Heterogeneity of PSA kinetics of the patient who progress to mCRPC was categorized into three types. PSA kinetics of the patients who respond to the initial ADT but immediately undergo PSA elevation in 7 months without deep PSA response (> 0.64 ng/mL) is categorized as “short and incomplete responder,” which predicts poorest prognosis. Others are categorized as “short responder” or “slow responder”. ADT, androgen deprivation therapy; mCRPC, metastatic castration‐resistant prostate cancer; mCSPC, metastatic castration‐sensitive prostate cancer; PSA, prostate‐specific antigen.

A model of PSA kinetics in mCSPC treated with initial ADT is proposed to predict the prognosis, in which PSA nadir > 0.64 ng/mL and time to nadir < 7 months are identified as poor prognostic factors (Figure 2) [84]. Furthermore, the clinical significance of PSA dynamics was demonstrated with ARSI and ADT, showing that achieving a much lower PSA nadir ≤ 0.02 ng/mL, a PSA response rate ≥ 99%, and time to nadir > 12 months is associated with a better prognosis [85]. Hence, a short duration of response to ADT, coupled with early nadir attainment at high PSA, is associated with poor prognosis, likely reflecting a higher proportion of androgen‐independent tumor cells [86]. However, it remains challenging to stratify patients who benefit from taxane‐based chemotherapy according to these risk stratification models [87].

Another challenge is the selection of appropriate subsequent therapies for patients with mCRPC after receiving ADT and ARSI and/or docetaxel. Because cross‐resistance reduces downstream effectiveness, intensification with available therapies, including alternative ARSI and cabazitaxel, along with the introduction of targeted therapies, is considered treatment strategies [88, 89, 90]. The CHAARTED2 trial has pointed to the benefits of combining cabazitaxel with an ARSI (abiraterone) compared with an ARSI alone, in terms of longer progression‐free survival and improved PSA response in mCRPC patients who have previously received ADT and docetaxel for mCSPC [91]. Importantly, the prevalences of genetic alterations are different depending on the stages of advancement. Aberrations in AR are infrequent in the early stage of PC, but AR pathway alterations and increased AR signaling commonly occur in advanced PC via amplification, gain‐of‐function mutations, or overexpression or increased transcription of AR. Genomic alteration of *PTEN* [89, 90] among the signatures associated with treatment resistance, PTEN loss drives PI3K‐AKT pathway hyperactivation, contributing to aggressive tumor behavior [92]. AKT inhibitors, by targeting this pathway, have shown potential clinical benefits in patients previously treated with ARSI when combined with standard‐of‐care therapies like docetaxel [93]. To validate this approach, ongoing clinical trials are assessing the efficacy of AKT inhibitors with docetaxel, aiming to improve outcomes in patients with PTEN‐deficient tumors [94].

Additionally, the optimization of treatment selection and sequence requires robust prospective studies designed to address the complexities of the disease. Approaches such as outcome‐adaptive randomized platform trials are particularly valuable, as they allow for the evaluation of treatment efficacy within predefined biomarker signatures, facilitating precision medicine [95, 96].

Moreover, the integration of machine learning is poised to revolutionize the analysis of clinical outcome data. By incorporating omics data and real‐world evidence, machine learning can uncover patterns and generate actionable insights to further refine treatment strategies. Together, these advancements hold the potential to transform the management of PC, improving both outcomes and the personalization of care [70, 97, 98, 99].

## 9 Conclusions

Taxane‐based chemotherapy remains essential in managing metastatic PC. Docetaxel, particularly in combination with ARSI and ADT, has demonstrated significant benefits for patients with high‐volume mCSPC. Furthermore, docetaxel has shown significant benefits for mCRPC if patients are taxane‐naive. Cabazitaxel offers an effective option for mCRPC patients who are exposed to docetaxel and ARSIs, particularly where cross‐resistance limits the efficacy of consecutive ARSI use. However, in the ARSI‐dominant era, evidence for optimal treatment sequencing for mCRPC remains limited. A deeper understanding of PC biology, along with a comprehensive collection of real‐world data, will be crucial to refining treatment strategies, improving therapeutic outcomes, and ensuring an optimal balance between efficacy and safety.

## Author Contributions

**Kazuhiro Suzuki:** writing – review and editing, conceptualization, writing – original draft. **Hideyasu Matsuyama:** writing – review and editing, writing – original draft, conceptualization. **Nobuaki Matsubara:** writing – review and editing, conceptualization, writing – original draft. **Hirotaka Kazama:** writing – review and editing, conceptualization, writing – original draft, supervision, data curation. **Fumiko Ueno:** writing – review and editing, conceptualization, writing – original draft, data curation, supervision. **Hirotsugu Uemura:** writing – review and editing, conceptualization, writing – original draft.

## Disclosure

K.S. has received honoraria from Sanofi KK, Astellas Pharma Inc., AstraZeneca PLC, Bayer AG, Janssen Pharmaceutical KK, and Takeda Pharmaceutical Co. Ltd. H.M. has received grants from Janssen Pharmaceutical KK and honoraria from Astellas Pharma Inc., Janssen Pharmaceutical KK, AstraZeneca plc, Bayer Yakuhin Ltd., and Merck Biopharma Co. Ltd. N.M. has received grants from Janssen Pharmaceutical KK, MSD KK, Bayer Yakuhin Ltd., Chugai Pharmaceutical Co. Ltd., Astellas Pharma Inc., AstraZeneca PLC, Bayer AG, Amgen Inc., Takeda Pharmaceutical Co. Ltd., Eli Lilly Japan KK, Eisai Co. Ltd., Roche Genentech Inc., Seagen Inc., Novartis Pharma KK, and AbbVie Inc.; consulting fees from Sanofi KK, Janssen Pharmaceutical KK, AstraZeneca plc, Eli Lilly Japan KK, Amgen Inc., Seagen Inc., Novartis Pharma KK, and Pfizer Japan Inc.; and honoraria from Sanofi KK. H.K. and F.U. are employees of Sanofi and may hold shares and/or stock options in the company. H.U. has received grants from AstraZeneca plc, Ono Pharmaceutical Co. Ltd., BMS KK, and Janssen Pharmaceutical KK; honoraria from Takeda Pharmaceutical Co. Ltd., BMS KK, Bayer AG, Sanofi KK, and Eisai Co. Ltd.; and has participated as a board member for AstraZeneca.

## Conflicts of Interest

The authors declare no conflicts of interest.
